# When – and whether – should we spay/neuter companion dogs

**DOI:** 10.1111/jsap.13894

**Published:** 2025-07-08

**Authors:** S. Romagnoli

**Affiliations:** ^1^ Department of Animal Medicine Production and Health University of Padova Legnaro Italy

## Abstract

The recent publication of guidelines for the control of reproduction in dogs and cats shows that a variety of non‐neoplastic and neoplastic conditions occur more commonly in spayed/neutered than entire dogs, and for several of such conditions the earlier the age at surgery – the higher the risk. In addition, unwanted behavioural changes may occur after gonadectomy rather than expected improvement in behaviour in some dogs. However, the fact that surgical gonadectomy increases the risk of any such condition does not mean that dogs should not be spayed/neutered any more. Gonadectomy still plays a major role in lowering the risk of mammary neoplasia and pyometra in bitches and of prostatic hypertrophy in male dogs. The evidence that gonadectomy influences the development of a condition is often conflicting, and it varies depending on the breed, sex and condition. This paper briefly illustrates current knowledge on the incidence of the above conditions in gonadectomised dogs, providing information on whether or not – and what is the best age at which – to perform gonadectomy in our companion dogs based on available scientific data.

## INTRODUCTION

Historically, gonadectomy has been the primary method of reproductive control in small animals, aimed at managing overpopulation and mitigating undesirable behaviours such as roaming, mounting, fighting and urine marking. Gonadectomy has been regarded as a key preventive measure against conditions such as pyometra and mammary tumours in bitches and queens and prostatic hyperplasia in male dogs. Also, it plays a role in preventing other less common conditions such as ovarian and testicular tumour, vaginal hyperplasia and prolapse in females and perianal gland disease and perineal hernia in males. Therefore, gonadectomy has gradually reached almost the same level of importance in clinical practice as vaccination in terms of caring for pet welfare although the frequency with which it is performed in clinical practice is country/culture‐dependent. The suggested age for gonadectomy has gradually reduced to prepubertal and even paediatric age based on the assumption that an earlier, technically simpler surgery would benefit the pet. Evidence accumulated over the past three decades shows that in fact this is not entirely true: a variety of non‐neoplastic and neoplastic conditions occur more commonly in spayed/neutered than entire dogs, and for several such conditions the earlier the age at surgery – the higher the risk. In addition, unwanted behavioural changes may occur after gonadectomy rather than expected improvement in behaviour in some dogs. This new information is causing a change of paradigm and the ongoing discussion among small animal practitioners is leading many to wonder what is the best age for gonadectomy and whether dog owners should not be advised to spay/neuter their animals any longer. This paper briefly illustrates current knowledge on the incidence of the above conditions in gonadectomised dogs providing information on whether or not – and what is the best age at which – to perform gonadectomy in our pets based on available scientific data and drawing on a recent set of guidelines produced for the control of reproduction in dogs and cats (Romagnoli et al., 2024).

## THE GOOD AND THE BAD OF GONADECTOMY

Advantages of gonadectomy may include prevention of infectious, inflammatory and neoplastic disorders of the reproductive tract, mammary glands and perineum; gonadectomy may mitigate unwanted behaviours such as roaming/mounting, urine marking and male fighting; also, it has been proposed to increase life expectancy (Banfield Pet Hospital Report, 2013; Barsanti & Finco, 1986; Beaudu‐Lange et al., 2021; DeTora & McCarthy, 2011; Gobello, 2021; Goericke‐Pesch et al., 2010; Hagman, 2023; Hart et al., 2016; Holst & Nilsson, 2023; Reichler, 2009; Root et al., 2018; Ruetten et al., 2021; Schneider et al., 1969; Williams et al., 2003; Zink et al., 2023).

However, possible benefits from gonadectomy may differ from our initial understanding. For instance, behavioural issues and life expectancy might not improve following gonadectomy and, in some cases, could even worsen (D’Onise et al., 2017; Maarschalkerweerd et al., 1997; Urfer et al., 2019). Furthermore, other disadvantages of gonadectomy have been increasingly reported in recent decades. An increased incidence of several types of neoplasia has been reported in gonadectomised male and female dogs (Anfinsen et al., 2011; Bell et al., 1991; Cooley et al., 2002; Dobson et al., 2002; Grüntzig et al., 2015; Knapp et al., 2000; Mochizuki et al., 2017; Oksanen, 1978; Polisca et al., 2016; Teske et al., 2002; Torres de la Riva et al., 2013; Zink et al., 2014). Also, obesity and urinary incontinence are causing increased concern among owners and veterinarians due to their chronicity and inherent increased risk of other diseases (Benka et al., 2023; McGreevy et al., 2005; Pegram et al., 2019; Spain et al., 2004).

The real extent of the impact of gonadectomy on each one of these diseases is still debatable as most of them are multifactorial and, in many instances, studies fail to take into consideration all factors involved. Conflicting reports have also been published concerning what is the best time at which to perform gonadectomy particularly relative to puberty. While recent research underlines the potential benefit of keeping domestic dogs entire, particularly for certain breeds and sexes, the body of evidence is still evolving. Definitive conclusions are hard to reach for the role of gonadectomy on most if not all of these conditions. However, it is certainly becoming increasingly difficult to present gonadectomy as a universal panacea for companion dogs as it used to be considered last century. As a consequence, advising clients on when – and whether – to have their companion dogs gonadectomised is becoming a challenge.

## WHAT IS THE ROLE OF GONADECTOMY IN CAUSING DISEASE?

Table 1 shows lifetime risks of various conditions (mast cell tumour, transitional cell carcinoma, osteosarcoma, lymphoma, haemangiosarcoma, prostatic carcinoma, urethral sphincter mechanism incompetence, orthopaedic diseases and obesity) in the entire canine population and their risk in gonadectomised male and female dogs.

**Table 1:** Incidence in the general (intact) canine population and risk in gonadectomized male and female dogs of conditions reported to occur following gonadectomy

Condition	Lifetime risk, intact canine population	Risk in gonadectomised male dogs	Risk in gonadectomised female dogs
Mast cell tumour	0.2% to 0.3% increases in some breeds (1, 3)	(1) **OR 3.5** *N* = 46/606 (M&F) *Vizlas* (2) **No effect** *N* = 10/249 *golden retrievers* (3) **OR 0.90 to 2.09** *N* = 512/971 depends on breed (4) **OR 1.15** *N* = 4415 (M&F)	(1) **OR 3.5** *N* = 75/825 (M&F) *Vizlas* (2) **RR= 4.46** *N* = 8/242 *golden retrievers* **DAG** (3) **OR 2.19 to 7.69** *N* = 590/889 (4) **OR 1.19** *N* = 4415 (M&F)
Transitional cell carcinoma	<1.0% (5)	(5) **OR 4.08** *N* = 258/426 (6) **OR 3.76** for lower urinary tract	(5) **OR 4.52** *N* = 726/1078 (6) **OR 3.76** for lower urinary tract
Osteosarcoma	0.2% to 9.0% (3, 7)	(7) **OR 1.4** *N* = 1689/3108 (8) **RR 1.4 to 3.8** 35/259 *Rottweiler* **IAG** (4) **OR 1.55** *N* = 842/67,943 (M&F) (9) **P < 0.0001** *N* = 37/550 (M&F) *Irish Wolfhound, Leonbergers*	(7) **OR 1.9** *N* = 1689/3108 (8) **RR 1.2 to 3.1** 51/338 *Rottweiler* **IAG** (4) **No effect** *N* = 842/67943 (M+F) (9) **P < 0.0001** *N* = 37/550 (M&F) *Irish Wolfhound, Leonbergers*
Lymphoma‐lymphosarcoma	<0.1% (10)	(10) **OR 2.8** *N* = 4040/11,677 all breeds (1) **OR 4.3** (*N* = 17/606) (M&F) *Vizlas* (11) **No effect** *N* = 3/245 *German shepherds* (12) **P = 0.007** *N* = 20/310 *golden retrievers* **DAG** (2) **P > 0.05** *N* = 17/247 *golden retrievers* **IAG**	(10) **OR 2.8** (*N* = 4040/11,677) all breeds (1) **OR 4.3** (*N* = 22/825) (M&F) *Vizlas* (11) **No effect** (*N* = 1/259) *German shepherds* (12) **P = 0.014** (*N* = 16/296) *golden retrievers* **DAG** (2) **No effect** *N* = 11/238 *golden retrievers*
Hemangiosarcoma	<2.0% (13)	(1) **No effect** *N* = 19/606 *Vizlas* (2) **No effect** (*N* = 10/389) *golden retrievers* (4) **No effect** *N* = 1904/67,943 (M&F) (14) **RR 1.63** *N* = 633/729,265 (M&F)	(1) **OR 9.0** *N* = 48/825 *Vizlas* **IAG** (2) **P = 0.01 DAG** *N* = 10/361 *golden retriever* (4) **OR 1.61** *N* = 1904/67,943 (M&F) (14) **RR 4.38** *N* = 633/729,265 (M&F)
Prostatic carcinoma	<1.0% (15 to 18)	(15) **OR 2.38** *N* = 31 (16) **No effect** *N* = 11 (17) **OR 4.3** *N* = 56 (19) **OR 3.8** *N* = 70 **DAG** (20) **No effect** *N* = 43	—
Urinary incontinence	<1.0% (21, 22)	(21) **No effect** *N* = 1027/109,428	(23) **HR 3.37, IAG** *N* = 493/72,971 (24) **HR 1.20, IAG** *N* = 1659 (25) **HR 2.23**, *N* = 3108/100,397
Orthopaedic diseases	5% (2)	(2) **P < 0.05** *N* = 18/221 HD, 10/248 CCL **IAG** (12) **P < 0.001** *N* = 7/270 CCL, ED *Labrador retrievers* (12) **P < 0.001** CCL (*N* = 14/312) HD (*N* = 25/281) *golden retrievers* **IAG** (26) **P < 0.05** *N* = 39/82, CCL, 11 breeds (27) **OR 1.21 to 1.68** *N* = 12,638/615,999 CCL, HD (28) **P < 0.001** *N* = 353/1672 (29) **RR 1.5** *N* = 76/346 IVDH **IAG** *Dachshunds* (32) **HR 1.2 to 2.7** *N* = 41/1508 M&F *golden retrievers* **IAG**	(2) **P < 0.05** *N* = 13/360 CCL **IAG** (12) **P < 0.001** *N* = 10/333 HD *Labrador retrievers* (12) **P < 0.001** CCL (*N* = 18/303) *golden retrievers* **IAG** (26) **P < 0.05** *N* = 73/119, CCL, 11 breeds (27) **OR 2.35** *N* = 25,014/627,682, CCL, HD (28) **P < 0.001** *N* = 442/2281 (29) **RR 2.12** *N* = 98/272 IVDH **IAG** *Dachshunds* (32) **HR 0.9 to 1.7** *N* = 41/1508 M&F *golden retrievers* **IAG**
Obesity	3% to 5% (30)	(31) **OR 1.5** *N* = 6073/17,963 M&F (32) **HR 1.2 to 2.7** *N* = 455/1508 M&F *golden retrievers* **IAG** (33) **OR 2.8 to 3.3** *N* = 17,852/45,732 M&F **DAG**	(31) **OR 1.5** *N* = 6073/17,963 M&F (32) **HR 0.9 to 1.7** *N* = 455/1508 M&F *golden retrievers* **IAG** (33) **OR 1.5 to 2.5** *N* = 17,852/45,732 M&F **DAG**

CCL Cranial cruciate ligament, DAG Direct association with age at gonadectomy (the older the age at gonadectomy the higher the risk), ED Elbow dysplasia, HD Hip dysplasia, IAG Inverse association with age at gonadectomy (the older the age at gonadectomy the lower the risk), IVDH Intervertebral disk herniation, M&F Males and females combined, *N* Number of cases of that specific condition/reference population of animals in that specific study

Data compilation is based on publications reporting significant risk/s for the selected condition/s expressed in **red** as odds ratio (**OR**), hazard ratio (**HR**) or relative risk (**RR**) values >1.0 or **P** values ≤0.05 reported from each study

Numbers between parentheses refer to the bibliography listed below: (1) Zink et al. (2014) Journal of the American Veterinary Medical Association 244, 309–319. (2) Torres de la Riva, et al. (2013) PLoS One 8, e55937. (3) White et al. (2011) Journal of the American Animal Hospital Association 47, 210–216. (4) Grüntzig et al. (2015) Journal of Comparative Pathology 152, 161–171. (5) Knapp et al. (2000) Urological Oncology 5, 47–59. (6) Norris et al. (1992) Journal of Veterinary Internal Medicine 6, 145–153. (7) Ru et al (1998) The Veterinary Journal 156, 31–39. (8) Cooley et al. (2002) Cancer Epidemiology Prevention Biomarkers 11, 1434–1440. (9) Anfinsen et al. (2011) Canadian Journal of Veterinary Research 75, 209–215. (10) Bennett et al. (2018) Journal of Veterinary Internal Medicine 32, 2054–2060. (11) Hart et al. (2016) Veterinary Medicine and Science 2, 191–199. (12) Hart et al. (2014) PLoS One 9, e102241. (13) Oksanen (1978) Journal of Comparative Pathology, 88 (4): 585–595. (14) Ware & Hopper (1999) Journal of Veterinary Internal Medicine 13, 95–103. (15) Bell et al. (1991) Journal of the American Veterinary Medical Association 199, 1623–1630. (16) Polisca et al. (2016) Theriogenology 85, 835–840. (17) Teske et al. (2002). Molecular and Cellular Endocrinology 197, 251–255. (18) Schrank & Romagnoli (2020) Animals 10, 85. (19) Sorenmo et al. (2003) Veterinary and Comparative Oncology 1, 48–56. (20) Obradovich (1987) Journal of Veterinary Internal Medicine 1, 183–187. (21) Hall et al. (2019) Journal of Small Animal Practice 60, 86–95. (22) Reichler & Hubler (2014) Reproduction in Domestic Animals 49 Suppl 2, 75–80. (23) Pegram et al. (2019) Journal of Small Animal Practice 60, 521–530. (24) Spain et al. (2004) Journal of the American Veterinary Medical Association 224, 380–387. (25) O’Neill et al. (2017) Journal of Small Animal Practice 58, 685–693. (26) Duval et al. (1999) Journal of the American Veterinary Medical Association 215, 811–814. (27) Witsberger et al. (2008) Journal of the American Veterinary Medical Association 232, 1818–1824. (28) Zink et al. (2023). Journal of the American Veterinary Medical Association 1, 1–9. (29) Dorn & Seath (2018) Canine Genetics and Epidemiology 5, 1–1433. (30) McGreevy et al. (2005) Veterinary Record 156: 695–702. (31) Lund et al. (2006) International Journal of Applied Research in Veterinary Medicine 4, 177. (32) Simpson et al. (2019) PLoS One 14, e0209131. (33) Benka et al. (2023) Journal of the American Veterinary Medical Association, 1–10

Information portrayed in the table is based on publications reporting significant risk/s for the selected condition/s expressed either as odds ratio, hazard ratio or relative risk values >1.0 or P values ≤0.05. The table may be used to help clinicians who are discussing reproduction control options with their clients. For example, when having to decide on performing orchiectomy in a young male golden retriever the table shows: increased risk of mast cell tumour (two studies), transitional cell carcinoma (two studies), osteosarcoma (two studies), lymphoma (three studies), prostatic carcinoma (three studies), orthopaedic problems (six studies) and obesity (three studies); also, two of the above studies were done on golden retrievers. For most of the cited conditions the reported risk is inversely associated with increasing age at gonadectomy with the exception of lymphosarcoma and obesity for which the opposite is true. If instead having to make the same decision about ovariectomy in a German shepherd bitch the number of publications is much lower showing only an increased risk of transitional cell carcinoma (one study) and haemangiosarcoma (one study); reports about the risk of orthopaedic diseases are conflicting (increased according to one study and decreased according to another, or breed is not specified in another study); there is no increased risk for obesity (one study) or the breed is not specified (one study); indications as to whether the age at gonadectomy plays a role are either missing or conflicting.

Conflicting evidence exists regarding the role of gonadectomy as a risk factor for mast cell tumour and prostatic carcinoma in male dogs and lymphosarcoma in female dogs. While studies on haemangiosarcoma in male dogs are also conflicting, a greater number suggest no increased risk associated with gonadectomy. Unanimous agreement is also present for osteosarcoma in males and mast cell tumours, haemangiosarcoma and urinary incontinence in females. Although agreement on a number of publications may not necessarily indicate reliability of study results, homogeneous results emerging from different studies may at least orient clinicians in advising clients.

Evidence‐based practice requires systematic evaluation of published data: checking methodology, number of animals, presence of a control group or any potential bias of the study protocol. In small animal reproduction, a systematic assessment of the scientific literature through a meta‐analysis has been done only for mammary tumours and urinary incontinence (Beauvais et al., 2012a, 2012b). The role played by gonadectomy in decreasing (mammary tumours) or increasing (urinary incontinence) the risk of these conditions was surprisingly regarded as weak (Beauvais et al., 2012a, 2012b). However, the finding of weak evidence does not necessarily mean that the tested hypothesis is not true as it may also mean that the methodology/animal numbers/comparability of groups of the considered studies may have not been appropriate for that specific purpose. Meta‐analyses are highly reliable statistical tools but require large number of studies with hundreds or thousands of patients, a feature which is easily achievable in human medicine but not as equally achievable in veterinary medicine, particularly small animal reproduction. The two above meta‐analysis papers (Beauvais et al., 2012a, 2012b) are certainly helpful in challenging current thinking; however, more elaborate studies are needed to further elucidate the role played by gonadectomy in these two conditions taking into account breed, age at gonadectomy with respect to puberty, diet before/after gonadectomy, lifestyle and general as well as urinary tract health.

Discussing the safety of gonadectomy with clients is especially important when the supporting studies involve large sample sizes – or focus on single breeds – as these factors can significantly influence the relevance and interpretation of the findings. For instance, few doubt about the role played by gonadectomy in increasing the risk of:

- lymphosarcoma and haemangiosarcoma in female golden retrievers (Bennett et al., 2018; Grüntzig et al., 2015; Hart et al., 2014; Torres de la Riva et al., 2013; Ware & Hopper, 1999; Zink et al., 2014);
- cranial cruciate ligament in male and female golden retrievers as well as in many large breeds (Duval et al., 1999; Hart et al., 2014; Witsberger et al., 2008);
- osteosarcoma in Rottweilers (Cooley et al., 2002; Grüntzig et al., 2015), in male golden retrievers (Hart et al., 2014; Torres de la Riva et al., 2013) and in large breeds in general (Anfinsen et al., 2011; Grüntzig et al., 2015; Ru et al., 1998);
- urinary incontinence in medium to large size bitches (O’Neill et al., 2017; Pegram et al., 2019; Spain et al., 2004).

In general, for breeds at risk of developing a neoplastic or orthopaedic disease (Bennett et al., 2018; Duval et al., 1999; Grüntzig et al., 2015; Witsberger et al., 2008) gonadectomy might increase this risk. Also, prostatic neoplasia is more common in neutered than entire dogs (Bell et al., 1991; Polisca et al., 2016; Sorenmo et al., 2003) and – although a clear relationship between neutering and development of prostatic neoplasia has not been established yet – the amount of evidence in favour of such a relationship is compelling (Romagnoli et al., 2024).

## IS THERE ENOUGH EVIDENCE TO JUSTIFY A RADICAL CHANGE OF PARADIGM, THAT IS, DO NOT SPAY/NEUTER ANYMORE?

The available data does not support a decision to stop spaying and neutering for all breeds of dogs. For many breeds there is still no available information and even for those for which odds ratios or relative risks for specific diseases are available, the initial risk in the entire population is generally so low that its doubling or any further increase may not raise any concern (Table 1) unless there is familiarity within the blood line of a specific dog. With some exceptions, small size breeds seem to be at lower risk of developing any of the above neoplasias (Grüntzig et al., 2015; Hart et al., 2020a) although there are conflicting reports with some showing an increased risk of neoplasia in small breeds following gonadectomy (Hoffman et al., 2013). Furthermore, some of the smaller breeds such as Corgi or Dachshunds may be at risk of developing orthopaedic problems (Bennett et al., 2018; Dorn & Seath, 2018). Therefore, even for small breed dogs it may be advisable to check the blood line history and discuss the surgical option at length with the owner.

## DOES AGE AT GONADECTOMY MATTER?

The age at which to perform spay/neuter surgery is a delicate choice. For many diseases for which gonadectomy is regarded as playing a role, performing the surgery at a very early age may actually increase the risk. Guidelines attempting to provide guidance on the age at which to perform gonadectomy have been published (Hart et al., 2020a, 2020b, 2023). Various studies show that paediatric (6 to 16 weeeks of age) and prepuberal (between 16 weeks and puberty) gonadectomy will increase the risk of orthopaedic diseases, particularly in medium to large and especially giant dog breeds, due to the fact that growth plate closure, especially of the radius and ulna, is delayed in these animals (Hart et al., 2014; Simpson et al., 2019; Torres de la Riva et al., 2013; Witsberger et al., 2008; Zink et al., 2023). Paediatric age gonadectomy is reported to further increase the risk of urinary incontinence (Pegram et al., 2019; Spain et al., 2004). Also, immaturity of external genitalia is a reported – albeit uncommon – side effect of gonadectomy performed prior to puberty which is thought to be due to the failure of the final stages of growth of external genitalia to occur. The recessed vulva which sometimes develops in prepubertally gonadectomised bitches may facilitate the development of perivulvar dermatitis predisposing to chronic vaginitis (Joshua, 1965; Salmeri, Bloomberg, et al., 1991; Salmeri, Olson, & Bloomberg, 1991). Because of all the above risks, gonadectomy prior to puberty is currently not recommended in companion dogs of all breeds (Romagnoli et al., 2024).

When spaying bitches soon after puberty, it is advisable to postpone surgery until the end of diestrus to allow progesterone priming of the reproductive system. Moreover, postponing surgery until anestrus will prevent the development of overt pseudopregnancy due to a sudden fall of progesterone concentration.

As concerns about evidence in favour of a role of gonadectomy in the development of canine prostatic adenocarcinoma increase, castration should probably not be regarded as the single best treatment for benign prostatic hypertrophy (Romagnoli et al., 2024). The decision should be taken while considering the role of age, the risk related to the breed, the (potential) reproductive value of the dog as well as the severity of the prostatic condition. In most dogs, benign prostatic hypertrophy responds well to a variety of treatments including progestogens, non‐steroidal anti‐androgens or long‐acting GnRH agonists; perhaps surgical gonadectomy could be considered for those cases that do not respond well to medical treatment. Although a final verdict on the role played by gonadectomy on the development of prostatic neoplasia cannot yet be made, the possibility of an early stage (microscopic) prostatic neoplasia developing faster following removal of androgen control, as hypothesised by Teske et al. (2002) should not be underestimated.

Gonadectomy is not an appropriate treatment for behavioural problems which should always be dealt with by a behavioural expert as spaying or neutering dogs – especially before puberty – may in some cases exacerbate behavioural issues (Romagnoli et al., 2024). Whenever gonadectomy is requested to eliminate a behavioural condition, a trial medical castration with a long‐acting GnRH agonist might be advisable as it would allow to check for the role of gonadal hormones on causing the behavioural problem.

The available information on the role played by gonadectomy in increasing the risk of some diseases is causing a major shift in paradigm for small animal practitioners around the world. Although dogs of many breeds and sizes can continue to be spayed/neutered depending on circumstances/health reasons/client convenience, the decision to routinely neuter all pets not intended for breeding can no longer be supported for all categories of animals. The option of leaving dogs unaltered unless there is a medical need for the dog or a constraint from the owner’s perspective should be evaluated. Gonad‐sparing spaying/neutering approaches such as hysterectomy and vasectomy are currently being re‐discovered (Zink et al., 2023) and should be re‐considered in small animal practice as they apparently have the same level of safety as classical spaying and neutering (although they do not eliminate reproductive behaviour). Owner’s reliability in managing an entire (or vasectomised/hysterectomised) dog should be assessed as these animals should undergo regular health checks to make sure mammary or prostatic conditions are diagnosed and treated at an early stage. In case these routine checks could not be reliably planned and adhered to, it would probably be in the best interest of dogs to be gonadectomised; however, it should be underlined that regular prostatic checks should be planned also for surgically gonadectomised dogs due to their higher risk of developing prostatic neoplasia. Routine orchiectomy and ovariectomy/ovariohysterectomy should be reserved for dogs of breeds at lower risk or for those animals for which the elimination of both reproduction and reproductive behaviour is necessary (Romagnoli et al., 2024).

From a practical standpoint, having to discuss potential health risks of classical spaying/neutering as well as the adoption of gonad‐sparing techniques will likely require longer consultations, necessitating adjustments in clinic scheduling and management. Straightforward routine recommendations are not possible any longer as advising clients on these issues will require taking into consideration age, sex, breed, animal lifestyle, purpose for which the animal is kept, age at which spay/neuter should be done as well as client’s perspectives and needs.

## Author contributions

**S. Romagnoli:** Conceptualization (equal); data curation (equal); formal analysis (equal); funding acquisition (equal); investigation (equal); methodology (equal); supervision (equal); visualization (equal); writing – original draft (equal); writing – review and editing (equal).

## Conflict of interest

No conflict of interest has been declared by the author.

## Data Availability

Data relative to this paper is available upon request.
